# Intramural hematoma and penetrating atherosclerotic ulcers of the aorta: uncertainties and controversies

**DOI:** 10.1590/1677-5449.180119

**Published:** 2019-07-12

**Authors:** Adamastor Humberto Pereira

**Affiliations:** 1 Hospital de Clínicas de Porto Alegre (HCPA), Universidade Federal do Rio Grande do Sul (UFRGS), Porto Alegre, RS, Brasil.

**Keywords:** acute aortic syndrome, intramural aortic hematoma, aortic hematoma, penetrating ulcers of aorta

## Abstract

The natural histories of intramural hematoma (IMH) and penetrating atherosclerotic ulcer (PAU) are highly variable as they may progress to aneurysm formation, rupture, or dissection, or even resolve, in the specific case of IMH. Imaging plays an increasingly important role in clinical and surgical management of IMH and PAU. In contrast to ulcer-like projections, images of intramural blood pools have not been widely reported in CT studies of patients with IMH. Understanding the imaging characteristics and the natural course of each of these entities would help clinicians and surgeons to identify patients at greatest risk for bad prognosis and may improve outcomes. This paper discusses the pathophysiology of these entities, the controversies regarding their natural history, and the prognostic factors that should be identified in CT scans.

## INTRODUCTION

The majority of publications consider intramural hematomas (IMH) and penetrating atherosclerotic ulcers (PAU) of the aorta to be variants of classical dissection, within the generic classification of acute aortic syndrome. The Stanford classification is used to guide management of all three clinical conditions.^1^ However, the pathophysiology and natural history of IMH and PAU are the subject of a great deal of debate and in many aspects are not related to classical dissection.

An IMH is defined as a hematoma of the aorta wall with no evidence of a media-intima entry rupture. However, in many cases, small intimal tears can be found during open procedures or using modern high-resolution imaging methods. Rupture of the vasa vasorum (VV) as cause of IMH is the subject of debate, and there is considerable evidence that these ruptures are a secondary phenomenon rather than the original cause of the IMH.^2^

A PAU is defined as erosion of the intima and the internal elastic membrane, with subsequent penetration of blood into the tunica media via atherosclerotic plaques. Clinical series in the literature cover small numbers of patients with PAU and IMH, and the natural history of these pathologies is the subject of much debate.^2^

Both entities occur in older patients than classical dissection, which has implications for initial clinical and radiological presentation and for the natural history of these aortic wall injuries.

## CHANGES TO THE AORTA WALL IN THE ELDERLY

To better understand the clinical and radiological presentation of PAU, IMH, and classical dissection, we must take into account the structural changes to the aorta that occur with aging. Compared with classical dissection, PAU and IMH occur in older patients who, consequently, have different parietal abnormalities. With aging, spaces appear between the elastic lamellae, caused by loss of the fine elastic fibers that join them, and fibrosis of the tunica media can be observed. These changes result in thickening and stiffening of the wall, which is exacerbated by arterial hypertension and the appearance of calcifications.

Elastin is synthesized by smooth muscle cells (SMC), which, in turn, are scarce in the major elastic arteries and reduce in number with aging. In addition to apoptosis of a considerable number of SMC, they separate from each other and their phenotype modifies to a state of senescence. The lost elasticity of the aorta results in rigidity, dilatation, stretching, and tortuosity.^3^^-^ 5

The correlation between tropoelastin synthesis by SMC and mRNA levels also changes. Expression of tropoelastin on the wall reduces with each decade of life beyond 60 years, which is the equivalent of a 94% reduction in elastogenic regenerative potential, when extrapolated to 40 years of age.^6^^-^ 8

These aging-related histological changes are observed with greater intensity in the external third of the tunica and are considered a fundamental part of the pathophysiologic mechanisms of aortic dissection and of IMH.^9^^,^ 10

## INTRAMURAL HEMATOMA AND THE ROLE OF THE VASA VASORUM

The adventitial VV are present in the large elastic vessels with more than 29 lamellae, and are thus found in greater numbers in the ascending aorta, the aortic arch, and the descending aorta, and in smaller quantities in the abdominal aorta.

The relationship between rupture of VV and IMH was first established in 1920 by Krukenberg^11^ and, since then, has been accepted as a possible cause of IMH. Intramural hematomas in the absence of PAU tend to be larger, sometimes compromising the ascending aorta, aortic arch, and descending aorta. The majority of publications accept the cause and effect relationship between rupture of VV and IMH, but there is scant evidence to confirm this interpretation. It seems difficult to explain that these small vessels, with low intraluminal pressure, can dissect large portions of the aorta and, in some cases, evolve to rupture of the artery wall. This mechanism is also unable to explain why the hematoma in IMH secondary to PAU tends to be limited, despite the existence of direct communication between the lumen and tunica media. Furthermore, Park et al. detected a 73% rate of intimal rupture among patients with type A IMH (in the ascending aorta) treated with surgery in whom a preoperative tomography did not detect any kind of defect.^12^

**Figure 1:**
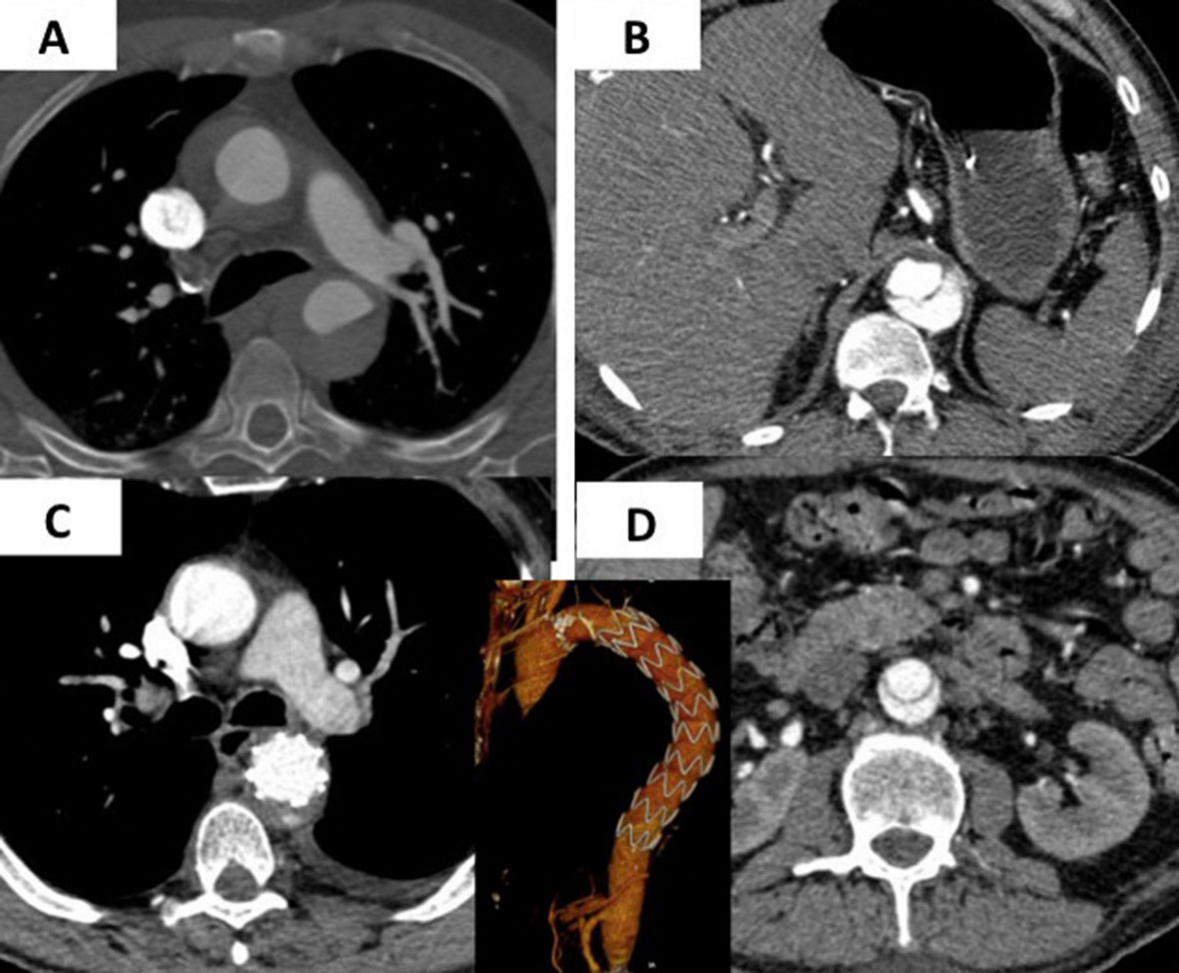
Angiotomography showing intramural hematoma (IMH) compromising the ascending aorta (A) in a 78-year-old patient with acute chest pain. In (B) the site of distal dissection can be seen close to the origin of the celiac trunk. In (C) complete resorption of the IMH by the ascending aorta has occurred within 30 days of placement of an endoprosthesis in the descending aorta, without compression of the true lumen distally (D). This case demonstrates why some IMH classified as Stanford type A are actually IMH originating in the descending aorta with retrograde progression of the hematoma.

In other words, a significant proportion of the IMH cases classified as type A using tomography were in fact cases of dissection with a small entry orifice that had sealed spontaneously. It is possible that these small intima-media tears are even more common, because, in cases of distal rupture with retrograde extension of the hematoma to the aortic arch and ascending aorta, these lesions are also erroneously classified as Stanford type A. It is no surprise, therefore, that intimal tears are not found during surgery in some cases (Figure 1).

More recently, higher-resolution tomography has increasingly demonstrated small intimal injuries and secondary lesions similar to ulcers (ulcer like projections) or intramural blood pools in serial examinations for follow-up of IMH, which are very often interpreted erroneously.

Chronic hypertension is a condition that is present in the majority of patients and is associated with occlusion of VV with neovascularization, increased arterial stiffness, and accelerated atherosclerosis. During a hypertensive crisis, medial ischemia can be aggravated by vasoconstriction of the VV. The most internal portions of the media continue to be fed by diffusion from the arterial lumen. This set of factors results in two regions in the tunica media with distinct characteristics: a more internal, and more elastic portion and a more rigid external portion. The difference in elastic modulus between these two regions sets up a difference in shear force at the interface.^13^^-^ 19 In the case of classical dissection, the tendency is for the true lumen to be compressed, because its external portion is more rigid. This change to the elastic modulus can also explain why the false lumen (less elastic and thinner) expands over time.

These altered mechanical elastic characteristics have been demonstrated experimentally in an animal model by resection of the adventitia to exclude the VV and ligature of the intercostal arteries (from which the VV originate in the descending aorta), provoking ischemia in the more external portions of the tunica media.^20^^,^ 21 After 2 weeks, it was observed that degradation of the elastic fibers had occurred in the external portion of the tunica media and spontaneous dissection took place in some of the animals. This experiment was replicated later with additional data collection after 4 and 8 weeks.^22^ This study demonstrated that, as the weeks passed, ischemia of the artery wall caused the extent of tunica media degeneration to increase, involving more internal portions. Thus, when there was advanced ischemia of the artery wall, the majority of the tunica media developed fibrosis. One interpretation of these findings is that progressive occlusion of the VV with aging, aggravated by hypertension and by the related atherosclerosis, can lead to diffuse degeneration of the tunica media. In these cases, when the intima ruptures, the cleavage plane is more external (adjacent to the adventitia) and the mural hematoma tends not to compress the true lumen. This could partially explain why, in many IMH, although rupture of the intima and large scale hematoma along the aorta can be seen, in the majority of cases, significant compression of the true lumen or occlusion of visceral branches are not observed. These findings may also explain why the mural hematoma that occurs in many cases of PAU is limited: the degenerated tunica media with extensive fibrosis does not allow the hematoma to propagate.

Recently, Osada et al. studied histopathological changes in patients with aortic dissection and confirmed the presence of degeneration and occlusion of the VV, associated with degradation of the elastic fibers and accumulation of extracellular matrix in the external third of the tunica media where dissection occurred.^23^

In contrast to a widely-held belief, compression of the true lumen cannot be explained by pressure differences alone, since experimental studies have demonstrated that neither systolic pressure nor pulse pressure are higher in the false lumen. It is clear that other factors, such as the size of the intimal tear and presence or absence of large reentry orifices with considerable retrograde flow, play important roles in compression of the true lumen.^24^^-^ 26

In the specific case of IMH, it has been demonstrated experimentally that peak stress on the tunica media (peak wall stress) is much greater than is observed in an artery without hematoma, which might explain progression to rupture in the direction of the lumen or frank dissection.^27^

## THE PATHOPHYSIOLOGIC SIGNIFICANCE OF ULCER LIKE PROJECTIONS AND BLOOD POOLS IN IMH

**Figure 2:**
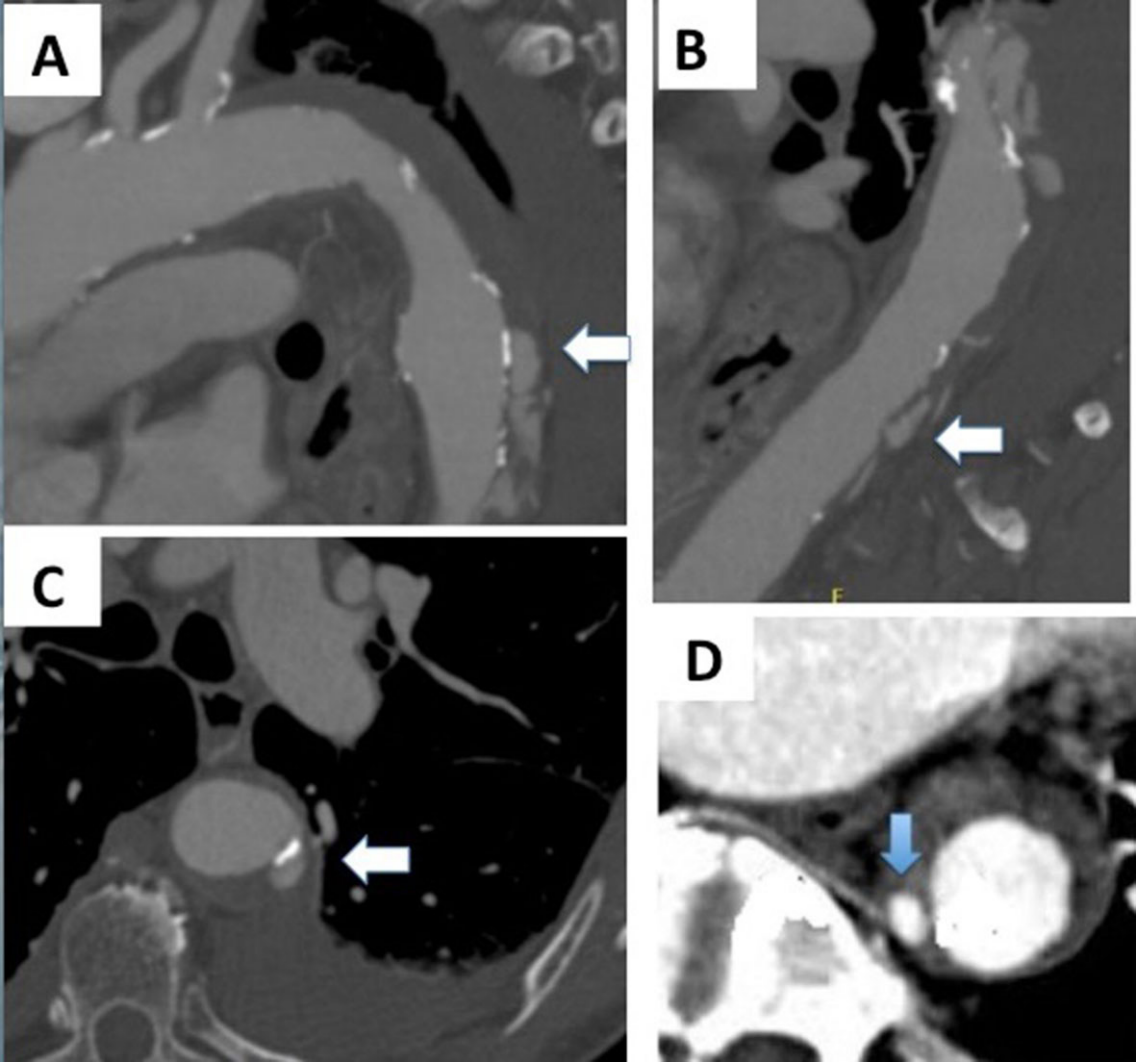
In (A), an ulcer like projection is shown. In (B), blood pools are arrowed. In (C), another ulcer like projection is shown, with adjacent pleural effusion. In (D), the relationship between a blood pool and an intercostal artery is shown.

In the case of a dissection with a small intimal-medial tear and no distal reentry to the true lumen, formation of the IMH may later be followed by sealing of the entry orifice. The appearance on X-ray would thus be an IMH with no detectable entry. In this case, the tunica media is subjected to very high stress, which has been demonstrated experimentally.^27^ Since the IMH develops in the more external portions of the media, it can progress with rupture of the origin of intercostal arteries, causing retrograde flow to the wall thus creating blood pools that are detected at the external surface of the hematoma. Blood pools can also be created by small intimal ruptures, appearing now in the internal surface of the hematoma. Other possibilities are: complete resorption of the hematoma, more significant ruptures in the direction of the lumen (creating ulcer like projections) or even dissection, formation of a pseudoaneurysm or frank rupture into the thoracic cavity (Figure 2).

Serial analysis of IMH patients with images typical of blood pools and ulcer like projections demonstrate that these injuries have completely different natural histories.^28^ Analysis of serial CT scans of patients with IMH shows that the blood pools that are present at onset or that appear over time are not associated with complications in the majority of cases. Wu et al. conducted a logistic regression analysis that identified the following factors as related to complications: aortic diameter exceeding 45 mm, location in the ascending aorta, and ulcer like projections.^28^

Prognosis was worse in the specific case of ulcer like projections (rupture into the intima) that were present at onset or that appeared during follow-up.^29^^-^ 31 Formation of pseudoaneurysm and progression to dissection or rupture occurred in 31% to 70% of cases and were manifest with greatest frequency in the ascending aorta.

There is a great deal of discussion in relation to descriptions of the natural history of these lesions and interpretations of CT images, since some publications, such as a recent European guideline and a 2018 UpToDate publication, state that IMH can progress to PAU or do not differentiate between PAU and ulcer like projections.^32^^,^ 33 As we have seen, by definition, PAU is characterized by erosion of the intima and media from atheromatous plaques. In contrast, ulcer like projections and some blood pools appear at onset or during progression of IMH and are caused by rupture into the lumen and, as such, develop in the opposite direction to PAU. Additionally, each of these lesions has its own specific clinical significance and course.

## PENETRATING ATHEROSCLEROTIC ULCER

Penetrating atherosclerotic ulcer of the aorta was first described by Shennan in 1934.^34^ As with IMH, PAU of the aorta is considered a variant of classical dissection, but with distinct clinical presentation and course.

**Figure 3:**
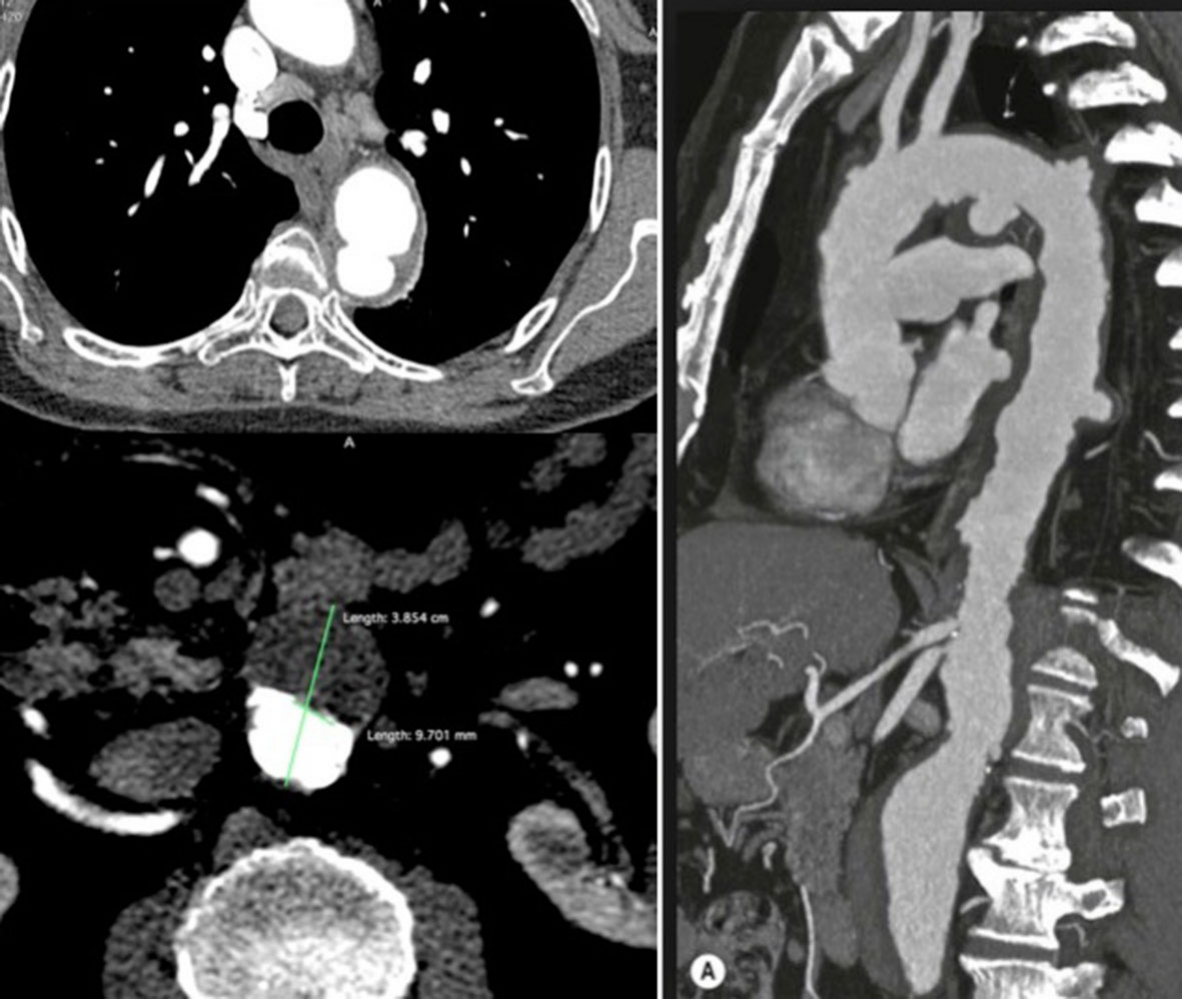
An 82-year-old patient with multiple penetrating ulcers of the aortic arch and descending aorta, associated with an abdominal aortic aneurysm. Measurements of the depth or size of the necks of ulcers are not reliable signs for indication of endovascular treatment.

Ulcerations of the thoracic aorta are relatively common in the elderly population, particularly after the seventh decade of life, but their true prevalence is yet to be determined (Figure 3). As with patients with IMH of the aorta, the majority of these patients are hypertensive. Deep ulcerations of the aorta wall can trigger symptoms similar to classical dissection and can occur in conjunction with secondary IMH (Figure 3). Ulcerations occur almost exclusively in the descending thoracic aorta, are frequently multiple, and can vary in size and depth; while abdominal aorta involvement is less common. Progression to rupture or frank dissection is associated with elevated morbidity and mortality.

### Natural history

The natural history of PAU of the aorta is not well known, even though PAU is one of the spectra of acute aortic syndrome.^35^^,^ 36

As is well explained by Ganaha et al.,^37^ there is confusion in the literature with relation to the behavior of PAU. It exhibits more aggressive behavior in symptomatic patients,^38^^,^ 39 in contrast with a more benign course in oligosymptomatic and, particularly, asymptomatic patients.^40^^,^ 41 Persistent or recurrent chest pain despite aggressive antihypertensives treatment, increased pleural effusion, and acute symptoms are negative prognostic factors, associated with greater risk of progression to aortic dissection or rupture. As is the case with IMH, ulcers that occur in the ascending aorta and the proximal portion of the descending aorta are linked with a larger number of complications. Possible explanations for this behavior include greater hemodynamic aggression to the artery wall at these points and predominance of elastin over collagen in the tunica media of the proximal aorta.^42^ Penetrating ulcers are responsible for around 2-7% of cases of acute aortic syndrome, with progression of the disease in both symptomatic and asymptomatic patients, justifying the need for control with serial imaging exams.^43^^,^ 44

Open or endovascular treatment is justified for symptomatic patients. However, in patients with asymptomatic PAU, it appears that conservative treatment is more appropriate, because there are not yet well-defined criteria that justify interventional treatment, whether for the thoracic or for the abdominal segments.^43^^,^ 45 Among these patients, the extent of penetration into the tunica media and the size of the neck of the injury are of questionable value as criteria for indicating surgery, as was demonstrated in the largest clinical series in the literature.^43^ In asymptomatic patients, indications for open or endovascular surgery are restricted to cases with evidence of pseudoaneurysm formation.

### Clinical status

The majority of ulcers that occur in elderly and hypertensive patients do not penetrate deeply into the tunica media and do not cause symptoms. Among these patients, episodes of microembolization may occur and trigger diagnosis. However, indication of endoprosthesis deployment in these situations is questionable, since ulcerations can occur in many different segments of the descending and abdominal aorta, which would require coverage of large extensions of the thoracoabdominal aorta, with obvious risks.

### Pathophysiology

In PAU, the atheromatous plaque extends deep into the artery wall, breaching the internal elastic membrane and penetrating the tunica media. When this penetration occurs, the tunica media is exposed to pulsating arterial flow, which can cause hemorrhage and IMH. In a Mayo Clinic series, 80% of cases of PAU were accompanied by IMH,^46^ but more recent series have reported much lower frequencies of associated IMH. This raises the question of whether many of these ulcerations might not actually have been ulcer like projections that developed during the course of an IMH and were incorrectly described as PAU.

### Imaging methods

A diagnosis of PAU of the aorta is established by anatomic criteria using CT, which shows a localized area of atheromatous plaque with focal ulceration and thickening of the aorta wall. The classic description on computed tomography is of a saccular, contrast-filled image that penetrates the aorta wall, surrounded by an IMH.

## CONCLUSIONS

There are few publications in the literature that deal with the pathophysiology of IMH and PAU, which causes confusion in relation to clinical and radiological interpretation of these injuries and of many aspects of their natural history and their true prevalence in the population. A number of different experimental studies have helped improve interpretation of clinical and radiological findings. The likelihood of prospective studies being undertaken in the near future is very small, and so the best course is to construct well-maintained registers, such as the International Registry of Aortic Dissection (IRAD), which has been accumulating information on acute aortic syndrome for two decades.^47^ It should be recognized that there are no well-established reporting standards for radiology or long-term medical follow-up with regard to IMH or PAU. These deficiencies should be corrected by our specialty societies in the form of specific registers, to promote better understanding of the changes affecting the aorta wall that are responsible both for the genesis and for the course of PAU and IMH. As Jean Martin Charcot (1825-1893) stated in *De l'éxpectation en médecine* 48: “Disease is very old and nothing about it has changed. It is we who change, as we learn to recognize what was formerly imperceptible”.
