# Supplementary material for: Intramural hematoma and penetrating atherosclerotic ulcers of the aorta: uncertainties and controversies
Source: J Vasc Bras. 2019 Jul 12;18:e20180119. doi: 10.1590/1677-5449.180119 (PMC6636911; doi:10.1590/1677-5449.180119)
Supplement: Translation

# Translation: Hematoma intramural e úlcera penetrante da aorta: incertezas e controvérsias

**Authors:** Adamastor Humberto Pereira

**Affiliations:** 1 Hospital de Clínicas de Porto Alegre (HCPA), Universidade Federal do Rio Grande do Sul (UFRGS), Porto Alegre, RS, Brasil.

**Keywords:** síndrome aórtica aguda, hematoma intramural da aorta, hematoma aórtico, úlcera penetrante da aorta

## Abstract

A história natural do hematoma intramural (HI) e da úlcera penetrante (UP) é muito variável, já que podem progredir para a formação de aneurisma, ruptura, dissecção, e podem até mesmo regredir, no caso específico do HI. Exames de imagem têm crescente papel no manejo clínico e operatório da UP e do HI. Ao contrário das projeções semelhantes a úlcera, achados de lagos sanguíneos não são relatados nos estudos tomográficos de pacientes com HI. O entendimento das características da imagem e do curso natural de cada uma dessas entidades ajudará clínicos e cirurgiões a identificar os pacientes com maior risco para um mau prognóstico e pode melhorar os desfechos. A fisiopatologia dessas entidades, as controvérsias no que se refere a suas histórias naturais e os fatores prognósticos das imagens tomográficas são discutidos neste artigo.

## INTRODUÇÃO

O hematoma intramural (HI) e a úlcera penetrante (UP) da aorta são considerados, pela maioria das publicações, como variantes da dissecção clássica dentro da denominação genérica de síndrome aórtica aguda. Nessas três condições clínicas, a classificação de Stanford é utilizada para orientar o tratamento^1^. Entretanto, a fisiopatologia e a história natural do HI e da UP são motivo de muitas dúvidas e não guardam relação com a dissecção clássica em muitos aspectos.

O HI é definido como hematoma da parede aórtica sem evidência de ruptura médio-intimal de entrada. Por outro lado, em muitos casos, pequenas rupturas da íntima podem ser evidenciadas durante procedimentos abertos ou pelos modernos métodos de imagem de alta resolução. A ruptura dos *vasa vasorum* (VV) como causa do HI é motivo de debate, e várias evidências sugerem que essa ruptura constitui fenômeno secundário e não a causa primordial do HI^2^.

A UP é definida como a erosão da íntima e da membrana elástica interna, com subsequente penetração do sangue na túnica média a partir de placas ateroscleróticas. As séries clínicas da literatura incluem um número restrito de pacientes com UP e HI, e a história natural dessas patologias é motivo de muito debate^2^.

Ambas as entidades ocorrem em pacientes mais idosos do que aqueles com dissecção clássica, o que tem implicações na apresentação clínica e radiológica inicial e na história natural dessas lesões da parede aórtica.

## ALTERAÇÕES DA PAREDE AÓRTICA EM IDOSOS

Para um melhor entendimento da apresentação clínica e radiológica da UP, do HI e da dissecção clássica, precisamos levar em consideração as alterações estruturais que ocorrem na aorta com o envelhecimento. Em comparação à dissecção clássica, a UP e o HI ocorrem em pacientes mais idosos e, portanto, com alterações parietais distintas. Com o envelhecimento, aparecem espaços entre as lamelas elásticas, devido à perda das finas fibras elásticas que as unem, e se identifica fibrose da túnica média. Essas alterações resultam em espessamento e aumento da rigidez da parede, que se agravam com a hipertensão arterial e o aparecimento de calcificações.

A elastina é sintetizada pelas células musculares lisas (CML), que por sua vez são escassas nas grandes artérias elásticas e diminuem em número com a idade. Além da apoptose de um número considerável das CML, estas se afastam uma das outras e modificam seu fenótipo para um estado de senescência. A perda da elasticidade da aorta resulta em rigidez, dilatação, alongamento e tortuosidade^3^^-^ 5.

A correlação entre a síntese da tropoelastina pelas CML e os níveis de mRNA também é modificada. A expressão da tropoelastina na parede diminui a cada década de vida após os 60 anos, o que representa uma redução de 94% do potencial elastogênico regenerativo, quando extrapolada para os 40 anos^6^^-^ 8.

Essas alterações histológicas ligadas ao envelhecimento são observadas com maior intensidade no terço externo da túnica e são consideradas como fundamentais no mecanismo fisiopatológico da dissecção aórtica e do HI^9^^,^ 10.

## HEMATOMA INTRAMURAL E O PAPEL DOS *VASA VASORUM*

Os VV adventiciais estão presentes nos grandes vasos elásticos com mais de 29 lamelas, e por essa razão estão em maior número na aorta ascendente, no arco aórtico e na aorta descendente, e em pequeno número na aorta abdominal.

A relação entre ruptura de VV e HI foi estabelecida pela primeira vez em 1920 por Krukenberg^11^ e, desde então, é aceita como possível causa do HI. O HI sem UP tende a ser mais extenso, às vezes comprometendo aorta ascendente, arco aórtico e aorta descendente. A maioria das publicações aceita essa relação de causa e efeito entre ruptura dos VV e HI, mas as evidências nesse sentido são escassas. Parece difícil explicar que esses pequenos vasos, com baixa pressão intraluminal, possam dissecar grandes porções da aorta e, muitas vezes, levar à ruptura da parede arterial. Esse mecanismo também não explica por que, no HI secundário à UP, o hematoma tende a ser mais restrito apesar de haver comunicação direta entre a luz e a túnica média. Por outro lado, a frequência detectada de ruptura da íntima em pacientes com HI do tipo A (aorta ascendente) submetidos a cirurgia e nos quais a tomografia não demonstrava qualquer defeito no pré-operatório foi de 73% no estudo de Park et al.^12^.

Em outras palavras, grande parte dos HI classificados como do tipo A pela tomografia eram na verdade casos de dissecção com pequeno orifício de entrada que havia selado espontaneamente. Talvez essas pequenas rupturas íntimo-mediais sejam ainda mais frequentes, porque, nos casos de ruptura distal com extensão retrógrada do hematoma para o arco aórtico e a aorta ascendente, essas lesões são também classificadas erroneamente como do tipo A de Stanford. Não é surpresa, portanto, que durante a cirurgia não sejam identificadas rupturas intimais (Figura 1).

**Figura 1:** Angiotomografia mostrando hematoma intramural (HI) comprometendo a aorta ascendente (A) em paciente de 78 anos com dor torácica aguda. Em (B) se identifica local de dissecção distal próxima à origem do tronco celíaco. Em (C) se observa a reabsorção completa do HI na aorta ascendente após 30 dias do implante de endoprótese na aorta descendente e sem compressão da luz verdadeira distalmente (D). Esse caso demonstra por que alguns HI classificados como tipo A de Stanford são na verdade HI originados na aorta descendente com progressão retrógrada do hematoma.

Os exames de tomografia de alta resolução mais recentes têm demonstrado com crescente frequência pequenas lesões intimais e lesões secundárias semelhantes a úlcera (*ulcer like projections*) ou lagos sanguíneos intramurais (*intramural blood pools*) nos exames seriados de acompanhamento dos HI, que muitas vezes são interpretados de maneira equivocada.

A hipertensão crônica é condição presente na maioria dos pacientes e se associa à oclusão dos VV com neovascularização, maior rigidez arterial e aterosclerose acelerada. Na crise hipertensiva, a isquemia da média pode se agravar devido à vasoconstrição dos VV. As porções mais internas da média permanecem nutridas por difusão a partir da luz arterial. Esse conjunto de fatores resulta em duas regiões com características distintas na túnica média: uma porção interna mais elástica e outra externa mais rígida. Devido às diferenças no módulo elástico das duas regiões, uma diferença na força de cisalhamento ocorre nessa interface^13^^-^ 19. No caso da dissecção clássica, a tendência é a compressão da luz verdadeira, pois a porção externa é mais rígida. Essa alteração no módulo elástico também poderia explicar por que a falsa luz (menos elástica e mais delgada) se expande ao longo do tempo.

Essas alterações das características elástico-mecânicas foram demonstradas experimentalmente em modelo animal pela ressecção da adventícia para exclusão dos VV e ligadura de intercostais (de onde os VV se originam na aorta descendente), provocando isquemia das porções mais externas da túnica média^20^^,^ 21. Após 2 semanas, se observou degradação das fibras elásticas das porções mais externas da túnica média e, em alguns animais, observou-se uma dissecção espontânea. Esse experimento foi replicado em nosso meio com dados adicionais após 4 e 8 semanas^22^. Nesse estudo, ficou demonstrado que, ao longo das semanas, a isquemia da parede arterial levou ao aumento da extensão da degeneração da túnica média, envolvendo agora as porções mais internas. Portanto, quando há isquemia avançada da parede arterial, a maior parte da túnica média evolui para fibrose. Uma interpretação desses achados é de que a oclusão progressiva dos VV com a idade, agravada pela hipertensão e pela aterosclerose associadas, pode levar à degeneração difusa da túnica média. Nesses casos, quando ocorre a ruptura da íntima, o plano de clivagem passa a ser mais externo (junto à adventícia) e o hematoma da parede tende a não comprimir a luz verdadeira. Isso poderia explicar em parte por que, em muitos HI, apesar de se identificar ruptura da íntima e grande hematoma ao longo da aorta, não se observa, na maioria dos casos, compressão significativa da luz verdadeira ou oclusão de ramos viscerais. Esses achados também poderiam explicar por que, em se tratando da UP, o hematoma mural, que aparece em muitos casos, é limitado: a túnica média degenerada e com fibrose extensa não permite que o hematoma se propague.

Recentemente, Osada et al. estudaram as alterações histopatológicas em pacientes com dissecção aórtica e confirmaram a presença de degeneração e oclusão dos VV, associada com degradação das fibras elásticas e acúmulo de matriz extracelular no terço externo da túnica média onde se desenvolveu a dissecção^23^.

Ao contrário do que muitos pensam, a compressão da luz verdadeira não pode ser explicada apenas por diferenças pressóricas, pois estudos experimentais demonstram que não se observa maior pressão sistólica ou pressão de pulso na falsa luz. É claro que outros fatores, como o tamanho da ruptura da íntima e a presença ou não de amplos orifícios de reentrada com grande fluxo retrógrado, têm papel importante na compressão da luz verdadeira^24^^-^ 26.

No caso específico do HI, já se demonstrou experimentalmente que o pico de estresse na túnica média (*peak wall stress*) é muito maior do que o observado na artéria sem hematoma, o que pode explicar a evolução para ruptura em direção à luz ou dissecção franca^27^.

## SIGNIFICADO FISIOPATOLÓGICO DAS PROJEÇÕES SEMELHANTES A ÚLCERA E LAGOS SANGUÍNEOS NO HI

No caso de uma dissecção com pequena ruptura íntimo-medial e sem reentrada distal para a luz verdadeira, o HI assim formado pode ser seguido do selamento do orifício de entrada. O aspecto radiológico seria então de HI sem entrada detectável. Nesse caso, a túnica média fica submetida a um estresse muito elevado, conforme já foi demonstrado experimentalmente^27^. Como o HI se estabelece nas porções mais externas da média, pode evoluir para desinserção da origem das intercostais e fluxo retrógrado para a parede, gerando os lagos sanguíneos detectados na face externa do hematoma. Os lagos sanguíneos podem ainda ser originados por pequenas rupturas intimais que aparecem agora na face interna do hematoma. As outras possibilidades são: reabsorção completa do hematoma, ruptura em direção à luz (gerando as projeções semelhantes a úlcera) ou dissecção continuada, formação de pseudoaneurisma ou ruptura franca para a cavidade torácica (Figura 2).

**Figura 2:** Em (A) se observa uma projeção semelhante a úlcera. Em (B) se identificam lagos sanguíneos. Em (C) se observa nova projeção semelhante a úlcera com derrame pleural adjacente. Em (D) se observa a relação de um lago sanguíneo com uma artéria intercostal.

A análise seriada de pacientes com HI com imagens típicas de lagos sanguíneos e projeções semelhantes a úlcera demonstra que essas lesões têm histórias naturais completamente diferentes^28^. A análise das tomografias seriadas desses pacientes com HI demonstra que os lagos sanguíneos presentes no início ou que apareciam ao longo do tempo não se associam a complicações na maioria dos casos. A regressão logística no estudo de Wu et al. apontou que os seguintes fatores estiveram relacionados a complicações: diâmetro aórtico maior do que 45 mm, localização na aorta ascendente e aparecimento de projeções semelhantes a úlcera^28^.

No caso específico das projeções semelhantes a úlcera (ruptura para a íntima) que estavam presentes no início do quadro ou que apareciam no seguimento, o prognóstico é pior^29^^-^ 31. A formação de pseudoaneurisma e a evolução para dissecção ou ruptura ocorrem em 31% a 70% dos casos e se manifestam com maior frequência na aorta ascendente.

Existe muita discussão em relação à descrição da história natural dessas lesões e à interpretação das imagens na tomografia, já que algumas publicações, como uma recente diretriz europeia e uma publicação do UpToDate de 2018, referem que o HI pode evoluir para UP ou não fazem diferenciação entre UP e projeções semelhantes a úlcera^32^^,^ 33. Por definição, a UP, como já vimos, caracteriza-se pela erosão da íntima e da média a partir de uma placa de ateroma. Por outro lado, as projeções semelhantes a úlcera e os lagos sanguíneos aparecem no início ou na evolução do HI e são decorrentes da ruptura para a íntima, ocorrendo, portanto, no sentido oposto ao da UP. Além disso, cada uma dessas lesões tem significado clínico e evolução particulares.

## ÚLCERA PENETRANTE

A UP de aorta foi inicialmente descrita por Shennan em 1934^34^. Assim como o HI, a UP da aorta é considerada uma variante da dissecção clássica, mas também com apresentação e evolução clínica distintas.

As ulcerações na aorta torácica são relativamente frequentes na população idosa, particularmente após a sétima década de vida, mas a real prevalência ainda está para ser determinada (Figura 3). Assim como ocorre com os pacientes com HI da aorta, a maioria dos pacientes são hipertensos. Ulcerações profundas na parede aórtica podem desencadear sintomas semelhantes aos da dissecção clássica e podem se associar a um HI secundário (Figura 3). As ulcerações associadas ocorrem quase exclusivamente na aorta torácica descendente, são frequentemente múltiplas, e podem variar em tamanho e profundidade; o envolvimento da aorta abdominal é menos comum. A evolução para ruptura ou dissecção franca está associada a morbimortalidade elevada.

**Figura 3:** Paciente de 82 anos com múltiplas úlceras penetrantes em arco aórtico e aorta descendente, associadas a aneurisma da aorta abdominal. As medidas da profundidade ou do tamanho do colo das úlceras não são sinais seguros para a indicação de tratamento endovascular.

### História natural

A história natural da UP de aorta não é bem conhecida, apesar de a UP representar um dos espectros da síndrome aórtica aguda^35^^,^ 36.

Como bem esclarece Ganaha et al.^37^, existe certa confusão na literatura quanto ao comportamento da UP. Observa-se um comportamento mais agressivo nos pacientes sintomáticos^38^^,^ 39, em contraste com um curso mais benigno nos pacientes oligossintomáticos e, principalmente, nos assintomáticos^40^^,^ 41. A dor torácica persistente ou recorrente, apesar do agressivo tratamento anti-hipertensivo, o aumento do derrame pleural e a presença de sintomas agudos são fatores prognósticos negativos, com maior risco de progressão para dissecção ou ruptura aórtica. Assim como ocorre no HI, as úlceras que ocorrem na aorta ascendente e na porção proximal da aorta descendente apresentam um maior número de complicações. As possíveis explicações para esse comportamento incluem uma maior agressão hemodinâmica na parede arterial nesses pontos e a preponderância da elastina sobre o colágeno na camada média da aorta proximal^42^. A UP é responsável por cerca de 2-7% dos casos de síndrome aórtica aguda, com progressão da doença tanto em pacientes sintomáticos quanto em assintomáticos, justificando a necessidade do controle seriado com exames de imagem^43^^,^ 44.

Nos pacientes sintomáticos, o tratamento aberto ou endovascular está justificado. Entretanto, em pacientes com UP assintomática, aparentemente o tratamento conservador parece ser mais apropriado, pois ainda não existem critérios bem definidos que justifiquem o tratamento intervencionista tanto no segmento torácico quanto no abdominal^43^^,^ 45. Nesses pacientes, a extensão da penetração na túnica média e o tamanho do colo da lesão são critérios discutíveis para a indicação cirúrgica, como ficou demonstrado na maior série clínica da literatura^43^. A indicação de cirurgia aberta ou endovascular em pacientes assintomáticos fica restrita aos casos com formação de evidente pseudoaneurisma.

### Quadro clínico

A maioria das úlceras que ocorrem nos pacientes idosos e hipertensos não penetra profundamente a túnica média e não causa sintomas. Nesses pacientes, episódios de microembolização podem ocorrer e conduzir ao diagnóstico. Todavia, a indicação para o implante de endoprótese nessas situações é discutível, pois as ulcerações podem ocorrer em vários segmentos da aorta descendente e abdominal, o que implicaria na cobertura de grandes extensões da aorta toracoabdominal, com riscos evidentes.

### Fisiopatologia

Na UP, a placa ateromatosa se estende profundamente na parede arterial, rompendo a membrana elástica interna e penetrando a camada média. Quando ocorre essa penetração, a camada média fica exposta ao fluxo arterial pulsátil, o que pode causar hemorragia e HI. Na série da Clínica Mayo, 80% dos casos de UP se acompanhavam de HI^46^, mas séries mais recentes relatam uma frequência muito menor de HI associado. A pergunta que se impõe é se muitas dessas ulcerações não seriam na verdade projeções semelhantes a úlcera que apareceram na evolução de um HI e foram erroneamente referidas como UP.

### Métodos de imagem

O diagnóstico da UP de aorta é estabelecido por critérios anatômicos no estudo tomográfico, evidenciado por uma área localizada de placa ateromatosa com ulceração focal e espessamento da parede aórtica. A descrição clássica na tomografia computadorizada é de uma imagem sacular preenchida por contraste que penetra a parede da aorta, circundada por um HI.

## CONCLUSÕES

As publicações que tratam da fisiopatologia do HI e da UP são escassas na literatura, o que gera confusão na interpretação clínico-radiológica dessas lesões, de muitos aspectos da sua história natural e da sua real prevalência na população. Vários estudos experimentais nos têm ajudado a interpretar melhor os achados clínicos e radiológicos. A possibilidade de que se realizem estudos prospectivos em futuro próximo é remota e, portanto, o caminho a seguir é a criação de registros bem conduzidos, como o *International Registry of Aortic Dissection* (IRAD), que há duas décadas vem acumulando informações sobre a síndrome aórtica aguda^47^. É preciso reconhecer que não há padrões bem estabelecidos na descrição radiológica (*reporting standards*) e no acompanhamento a médio e longo prazo no que se refere ao HI e à UP. Essas deficiências precisam ser corrigidas por nossas sociedades de especialidade com registros específicos, para um melhor entendimento das alterações parietais da aorta responsáveis tanto pela gênese como pelo comportamento evolutivo da UP e do HI. Como dizia Jean Martin Charcot (1825-1893) em *De l'éxpectation en médecine* 48: “A doença é muito antiga e nada nela mudou. Somos nós que mudamos quando aprendemos a reconhecer o que antes não era percebido (*Disease is very old and nothing about it has changed. It is we who change, as we learn to recognize what was formerly imperceptible*).
